# Ruminal inocula with distinct fermentation profiles differentially affect the *in vitro* fermentation pattern of a commercial algal blend

**DOI:** 10.3389/fvets.2024.1346683

**Published:** 2024-03-11

**Authors:** Cátia S. C. Mota, Margarida R. G. Maia, Inês M. Valente, Ana R. J. Cabrita, António J. M. Fonseca

**Affiliations:** ^1^REQUIMTE, LAQV, ICBAS, School of Medicine and Biomedical Sciences, University of Porto, Porto, Portugal; ^2^REQUIMTE, LAQV, Department of Chemistry and Biochemistry, Faculty of Sciences, University of Porto, Porto, Portugal

**Keywords:** algal blend, dairy cow, inocula, *in vitro*, fermentation, rumen

## Abstract

The *in vitro* rumen batch technique is widely used for screening novel feed sources; however, it remains unclear to what extent the *in vitro* fermentability of non-conventional feed sources is affected by non-adapted ruminal inocula. Thus, in this study, we evaluated the effects of distinct ruminal inocula on the *in vitro* fermentation parameters of a sustainable non-conventional feed, a commercially available algal blend composed of microalgae (*Chlorella vulgaris* and *Nannochloropsis oceanica*) and seaweeds (*Ulva* sp. and *Gracilaria gracilis*). First, four late-lactation Holstein cows were fed four forage-based diets varying only in the proportions of basal forage (100% corn silage, 70% corn silage and 30% haylage, 30% corn silage and 70% haylage, and 100% haylage) in a 4 × 4 Latin square design with the last square omitted. After 3 weeks of adaptation, haylage-based diets resulted in ruminal fermentation parameters distinct from those promoted by corn silage-based diets, as reflected in increased pH, ammonia-N contents, and acetate proportions. Individual ruminal fluids derived from each of the four diets were further used as inocula in *in vitro* incubations. Here, a 1:1 mixture of corn silage and haylage was supplemented with 0, 5, 10, or 15% algal blend and incubated with each inoculum for 24 h in a 4 × 4 factorial design. Total gas and methane production decreased with inocula from cows fed haylage-based diets and with increasing algal blend supplementation levels. The fermentation pH increased and the ammonia-N contents decreased with inocula from cows fed haylage-based diets; however, these parameters were not affected by algal blend inclusion levels. The interaction between the ruminal inoculum source and the algal blend supplementation level affected the total volatile fatty acids (VFA) and the proportions of most individual VFA. Total VFA production decreased with increasing algal supplementation levels, particularly with inocula from cows fed 30% corn silage and 70% haylage; the acetate, propionate, and valerate proportions were only affected by algal blend levels under incubation with 100% corn silage inocula. Overall, our findings highlight the importance of the ruminal inoculum source when assessing the fermentability of non-conventional feed as well as the potential of the algal blend as a natural modulator of ruminal fermentation.

## 1 Introduction

The number of challenges facing livestock production systems is increasing at an unprecedented rate. There is a growing demand for animal-derived foods in terms of both quality and quantity, particularly in developing countries. Accordingly, there is a need to ensure food and nutrition security to sustain the growth of the human population (1) while simultaneously counteracting the pressures on available resources and the effects of climate change. More sustainable livestock systems, in particular those relating to ruminants, are needed to enhance animal productivity, health, and welfare and increase food production while reducing the negative impact of farms on the environment and food–feed competition (2). Non-conventional (novel or underexploited) feeds are of particular interest as they can effectively contribute to improving ruminant-derived food production, which is in line with the projected transition toward a circular bio-based economy in the European Union. Direct-fed microbial, microalgae, seaweeds, aquatic plants (e.g., duckweed), oilseeds (e.g., camelina), tropical tree and shrub leaves (e.g., cassava, *Leucaena* sp., and *Flemingia* sp.), and fruit and vegetable by-products have all been suggested as potential non-conventional ruminant feed resources given their low arable land and water requirements as well as their potential to reduce enteric methanogenesis and promote healthier ruminant-derived foods and more sustainable production systems (3–6).

Feed evaluation studies are needed to fully assess the potential of non-conventional feeds as ingredients or additives in ruminant diets. Studies evaluating production responses and *in vivo* digestibility require a large number of animals and amounts of feed, require an extensive investment in labor and time, are expensive (7), and raise ethical issues regarding the use of untested non-conventional feeds and the use of animals for experimental purposes. Consequently, *in vitro* methodologies (e.g., Tilley and Terry method, Ankom Daisy system, Hohenheim gas test, batch culture technique, and rumen simulation technique) are increasingly being used for evaluating the degradability and fermentability of novel feeds, ingredients, and supplements. Despite providing useful data, these methodologies require ruminal microbial inocula to recreate a ruminal fermentation environment and fail to mimic the complete ruminal digestive process (7).

The need for ruminal fluid inocula adds a source of variation owing to differences within and between animals used as rumen digesta donors, which can affect *in vitro* fermentation results. To reduce this source of variation and error, the ruminal inocula used for *in vitro* studies should be representative of the *in vivo* microbiota composition and activity (7, 8), and true replicates must be employed (9). Dietary composition and nutrient availability are the factors that most contribute to variation in rumen microbiota composition and activity (8, 10). Accordingly, the diets of rumen inoculum donors should be similar to those used in *in vitro* feed evaluation systems (8, 11). However, this finding is difficult to achieve when assessing non-conventional ingredients or additives as feed resources for ruminants, and most *in vitro* studies use non-adapted ruminal inocula.

Feeding strategies, such as varying the neutral detergent fiber-to-rapidly fermentable carbohydrate ratio [acidogenicity value concept; (12, 13)] or the physically effective neutral detergent fiber content (14) of diets, can effectively modulate rumen function without negatively affecting the health and welfare of animals. Thus, we hypothesized that ruminal inocula with distinct fermentation profiles could be attained by altering the proportion of basal forage in the diets of the ruminal inoculum donors from corn silage to haylage, two of the most commonly used forages in dairy feeding, and that these changes would affect the fermentation profile of non-conventional feeds after 24 h of *in vitro* incubation with non-adapted ruminal inocula. In this context, increasing levels (0, 5, 10, and 15%, on a dry matter [DM] basis) of a sustainable, non-conventional feed, a commercially available algal blend composed of two microalgal (*Chlorella vulgaris* and *Nannochloropsis oceanica*) and two seaweed (*Ulva* sp. and *Gracilaria gracilis*) species, were added to a basal substrate (corn silage: haylage, 1:1 DM basis), and each mixture was separately incubated with individual ruminal inocula derived from each of the four diets containing different proportions of corn silage and haylage. The effects on gas and methane production and the fermentation profile were subsequently evaluated after 24 h of *in vitro* batch incubation.

## 2 Materials and methods

### 2.1 Ethics statements

All procedures involving animals were approved by the Animal Ethics Committee of the School of Medicine and Biomedical Sciences, University of Porto (ICBAS-UP) and licensed by the Portuguese General Directorate of Food and Veterinary Affairs (permit #0421/000/000/2021). All the procedures were performed by trained scientists (FELASA category C) in accordance with the European Union Directive 2010/63/EU relating to the protection of animals used for scientific purposes.

### 2.2 *In vivo* trial

#### 2.2.1 Animals, diets, and management

Four healthy, primiparous Holstein cows, pregnant and in late lactation (507 ± 17.8 days in milk), were used in the trial, which was conducted between July and September 2022. Each animal was fitted with a ruminal cannula (10 cm in diameter; Bar Diamond Inc., Parma, ID, USA). The cows were housed at the Vairão Agricultural Campus of ICBAS-UP (Vila do Conde, Portugal) in contiguous individual boxes (10–11.5 m^2^), which allowed and promoted contact, grooming, and socializing and had continuous access to drinking water and mineral salt blocks. The cows had access to an outdoor paddock from 12:00 h to 15:00 h daily.

The animals were randomly assigned to four experimental diets in an incomplete 4 × 4 Latin square design (with the last period omitted). Each experimental period lasted for 3 weeks. The diets were formulated to promote changes in ruminal function by varying the proportions of basal forage (corn silage and/or haylage) but keeping the concentrations of the remaining ingredients unchanged (chopped barley straw, commercial concentrate, and soybean meal) (Table 1). The experimental diets were named according to the proportion of the basal forage used as follows: 100% corn silage, 100CS; 70% corn silage and 30% haylage, 70CS30HL; 30% corn silage and 70% haylage, 30CS70HL; and 100% haylage, 100HL.

**Table 1:** Ingredient and chemical composition (% dry matter [DM]) of the experimental diets and the amount of feed offered (kg DM/day).

	Diets^1^			
	100CS	70CS30HL	30CS70HL	100HL
Ingredient composition				
Corn silage	53.5	37.3	15.8	0.0
Haylage	0.0	16.5	38.3	54.2
Chopped barley straw	15.5	15.4	15.3	15.3
Commercial concentrate	25.9	25.7	25.5	25.5
Soybean meal	5.1	5.1	5.0	5.0
Chemical composition				
Dry matter, %	44.8	50.7	61.1	72.2
Ash	4.7	5.6	6.8	7.7
Crude protein	13.3	13.8	14.6	15.1
Ether extract	2.9	2.7	2.3	2.1
Neutral detergent fiber	44.6	46.7	49.4	51.5
Starch	24.9	18.9	10.8	4.9
NSC^2^	34.5	31.2	26.8	23.6
NSP^3^	9.6	12.4	16.0	18.7
Amount offered, kg DM/day	17.5	17.6	17.7	17.8

^1^Diets are named according to the proportions (DM basis) of corn silage (CS) and haylage (HL) used as the basal forage; 100CS, 100% CS; 70CS30HL, 70% CS and 30% HL; 30CS70HL, 30% CS and 70% HL; 100HL, 100% HL.

^2^NSC, non-structural carbohydrates = 100 – (neutral detergent fiber + crude protein + ether extract + ash).

^3^NSP, non-starch polysaccharides = non-structural carbohydrates – starch.

Corn silage was prepared in September 2021. Ensilage was performed in a bunker silo with the use of a silage additive. Haylage (based on ryegrass) was prepared at the end of April 2022. After harvesting followed by a wilting period, the grass was baled in big bales and a silage additive was used.

The cows were milked twice daily at 08:00 h and 18:15 h. At each milking session, the animals received and consumed 2.5 kg of commercial concentrate plus 0.5 kg of soybean meal. Basal forage (corn silage and/or haylage) mixed with barley straw was fed to the animals twice a day (08:30 h and 18:45 h). Orts were collected and weighed daily before morning milking and feeding. Every day during the last week of each experimental period, DM intake was determined for each animal, and samples of experimental diets and orts were collected for chemical composition analysis. At the end of each experimental period, the diets were exchanged between cows, and a new 3-week experimental period began.

#### 2.2.2 *In vivo* sample collection

On the last day of each experimental period, before morning milking and feeding, ruminal contents were collected from the four quadrants of the rumen into pre-heated (39°C) thermos containers and the pH was immediately measured. The ruminal samples were quickly transported to the laboratory to ensure the viability of the ruminal microbial population and individually filtered through four layers of cheesecloth under anaerobic conditions. Samples of the strained ruminal fluid were collected for ammonia-N and volatile fatty acid (VFA) analyses. Ruminal fluid samples from the second and third experimental periods were further used as inocula in the short-term *in vitro* incubation trial.

### 2.3 *In vitro* trial

#### 2.3.1 Algal blend, substrates, and *in vitro* incubation

To assess the effect of the ruminal inocula on the *in vitro* fermentation of a novel feed, the basal forages (corn silage and haylage) of the *in vivo* trial were used as substrates. The basal substrates were dried at 65°C for 48 h, ground through a 1-mm sieve, and stored at room temperature until incubation. The novel feed evaluated was a commercially available algal blend (Algaessence® feed; Algaessence is a cobranding of microalgae [Allmicroalgae, Pataias, Portugal] and seaweeds [ALGAplus, Ílhavo, Portugal] producers) composed of two microalgal species (*C. vulgaris* and *N. oceanica*) and two seaweed species (*Ulva* sp. and *G. gracilis*) produced autotrophically in closed tubular photobioreactors and integrated multitrophic aquaculture (IMTA) systems, respectively. Algaessence, commercialized as a spray-dried powder in sealed light-protected bags, was kept at room temperature in the dark until incubation. The commercial algal blend presented (on a DM basis) a high ash content (22.1%); moderate crude protein (36.2%), non-starch polysaccharide (17.1%), and non-structural carbohydrate (14.2%) contents; and a low starch content (2.90%). Palmitic (C16:0, 12.2 mg/g), palmitoleic (C16:1 *cis*-9, 5.69 mg/g), linolenic (C18:3 *n*-3, 5.13 mg/g), oleic (C18:1 *cis*-9, 3.91 mg/g), linoleic (C18:2 *n*-6, 3.62 mg/g), and eicosapentaenoic (EPA) (C20:5 *n*-3, 3.52 mg/g) acids were the main fatty acids present in the blend (Table 2).

**Table 2:** Chemical composition (% dry matter) and selected fatty acids (mg/g dry matter) of the basal substrates (corn silage and haylage) and the algal blend used in the 24-h *in vitro* incubations.

	Corn silage	Haylage	Algal blend
Chemical composition			
Ash	2.41	9.73	22.1
Crude protein	5.30	11.3	36.2
Neutral detergent fiber	41.9	59.7	19.9
Ether extract	3.31	1.87	4.67
Starch	41.4	n.d.	2.90
NSP^1^	47.1	17.4	17.1
NSC^2^	5.68	17.4	14.2
Fatty acids			
C16:0	4.78	2.25	12.2
C16:1 *cis*-9	0.036	0.145	5.69
C18:1 *cis*-9	3.94	0.343	3.91
C18:2 *n*-6	9.45	1.61	3.62
C18:3 *n*-3	1.27	3.08	5.13
C18:4 *n*-3	n.d.	n.d.	0.660
C20:4 *n*-6	n.d.	n.d.	2.53
C20:5 *n*-3 (EPA)	n.d.	n.d.	3.52
C22:5 *n*-3	n.d.	n.d.	0.124

^1^NSC, non-structural carbohydrates = 100 – (neutral detergent fiber + crude protein + ether extract + ash).

^2^NSP, non-starch polysaccharides = non-structural carbohydrates – starch.

EPA, eicosapentaenoic acid; n.d., not detected.

Four experimental treatments (a 4 × 4 experimental design) were devised based on a basal substrate (corn silage and haylage, 1:1 DM basis) supplemented on top with incremental algal blend levels (up to 15%) and incubated for 24 h in batch culture systems with the four rumen inocula collected in the *in vivo* trial. Briefly, 250 mg of corn silage and 250 mg of haylage were placed in 150-mL serum bottles (Sigma-Aldrich Inc., St. Louis, MO, USA) and supplemented with (DM basis) 0% (A0), 5% (A5), 10% (A10), or 15% (A15) algal blend. Individual strained ruminal fluids (100CS, 70CS30HL, 30CS70HL, or 100HL) were mixed with the Mould buffer solution (8) at a 1:4 (*v*/*v*) ratio and kept at 39°C under O_2_-free CO_2_. In total, 50 mL of buffered ruminal inoculum was added, under an O_2_-free CO_2_ stream, to the serum bottles containing the experimental treatments. The bottles were sealed with butyl rubber septa and aluminum caps (Sigma-Aldrich Inc., St. Louis, MO, USA) and placed in a water bath at 39°C for 24 h under orbital agitation. Bottles without substrate (corn silage, haylage, or algal blend) but containing buffered ruminal inocula (blank samples) were incubated in parallel. All experimental treatments were incubated in duplicate per inoculum and per each of two incubation runs.

#### 2.3.2 *In vitro* sample collection

After 24 h of incubation, the fermentation was immediately halted by placing the bottles in an iced water bath for 30 min. The bottles were then gradually warmed to 25°C, and the total gas production was measured using a pressure transducer (Bailey & Mackey Ltd., Birmingham, UK), as described by Maia et al. (6). The fermentation gas produced was collected with an air-tight glass syringe (SGE International PTY Ltd., Ringwood, Victoria, Australia), and the methane composition was determined by gas chromatography (15). Subsequently, bottles were opened, and the pH of the fermentation medium was immediately measured (GLP 22+ pH meter; Crison, Barcelona, Spain). Samples of the fermentation medium were further collected and kept at −20°C for the analysis of VFA production and ammonia-N contents.

### 2.4 Analytical determination

#### 2.4.1 Proximate composition analysis

The proximate composition of ground (through a 1-mm sieve) dietary ingredients, orts, forages used as *in vitro* substrates and the algal blend were analyzed according to previously described official methods (16). All the samples were assessed for DM (method 934.01), ash (method 942.05), ether extract (method 920.39), and Kjeldahl N (method 954.01) contents. The crude protein content was calculated as N × 6.25 (method 990.03). In addition, the neutral detergent fiber (with α-amylase and without sodium sulfite) content (17, 18) was determined in all samples and expressed without residual ash. Owing to the small size of the microalgal species present in the algal blend (<25 μm in diameter), the filtration step in neutral detergent fiber determination was modified; that is, glass microfiber filters (Whatman GF/A, 1.6-μm porosity, Merck KGaA, Darmstadt, Germany) were used instead of P2 crucibles (40–100-μm porosity). The starch content was determined in 0.5-mm ground samples after hydrolysis to glucose and reaction with the glucose oxidase–peroxidase (GOPOD) reagent (Megazyme, Wicklow, Ireland) and determined spectrophotometrically at 505 nm (Synergy HT Multimode plate reader, BioTek Instruments, Bad Friedrichshall, Germany) (19). All analyses were performed in duplicate.

#### 2.4.2 Fatty acid analysis

The fatty acids in corn silage, haylage, and the algal blend were transesterified by acid-catalyzed methylation (20) using nonadecanoic acid (C19:0, Matreya LLC, State College, PA, USA) as the internal standard. The resulting fatty acid methyl esters were analyzed using a Shimadzu GC-2010 Plus gas chromatograph (Shimadzu Europe GmbH, Duisburg, Germany) equipped with a capillary column (Omegawax 250, 30 m × 0.25 mm internal diameter, 0.25 μm film thickness; Supelco, Bellefonte, PA, USA) and a flame ionization detector, as described by Mota et al. (21). The identification of fatty acids was done by comparison with commercial standards (Supelco 37 Component FAME Mix, BAME Mix, PUFA No. 1, PUFA No. 2, PUFA No. 3, Sigma-Aldrich Co. LLC; GLC-110 Mixture, Matreya LLC) and quantified based on the internal standard (C19:0). Analyses were run in duplicate.

#### 2.4.3 Fermentation end-products

Total VFA production and the individual VFA profile of the strained ruminal fluid and fermentation medium were determined as previously described (15). Briefly, 1 mL of ruminal fluid or fermentation medium and 0.25 mL of 25% ortho-phosphoric acid solution with 16 mM 3-methylvaleric acid (internal standard; Sigma-Aldrich, Inc.) were mixed and centrifuged at 19,800 × *g* for 15 min at 4°C. The supernatant was collected and analyzed using a gas chromatograph (Shimadzu GC-2010 Plus, Shimadzu Corporation, Kyoto, Japan) equipped with a flame ionization detector and a capillary column (HP-FFAP, 30 × 0.25 mm; 0.25 μm film thickness; Agilent Technologies, Santa Clara, CA, USA). Individual VFA were identified by comparing their retention times with that of a commercially available standard (Volatile Free Acid Mix, Sigma-Aldrich, Inc.) and quantified using the internal standard.

Strained ruminal fluid and fermentation medium samples were also analyzed for ammonia-N content following the method of Chaney and Marbach (22). Briefly, 1 mL of sample and 0.25 mL of 25% ortho-phosphoric acid solution were homogenized and centrifuged at 12,000 × *g* for 20 min at 4°C; a volume of 40 μL of the supernatant was added to 40 μL of water, 2.5 mL of phenol solution, and 2 mL of alkaline hypochlorite solution. The mixture was homogenized and incubated at 37°C for 10 min. The absorbance was subsequently read in a spectrophotometer at 550 nm (Synergy HT). The ammonia-N content was quantified using a calibration curve of an ammonia solution (0–32 mg/dL).

### 2.5 Statistical analysis

Statistical analysis of DM intake averaged by period and fermentation data for the *in vivo* and *in vitro* trials was performed with SAS software (2023; SAS OnDemand for Academics, SAS Institute Inc., Carry, NC, USA) using the general linear model procedure. The *in vivo* data were analyzed according to a Latin square design, with the model including the fixed effects of cow, period, and diet, as well as the residual error. The *in vitro* data were analyzed according to a 4 × 4 factorial design. The model included the fixed effects of cow, period, ruminal inoculum source, algal blend inclusion level, the interaction between ruminal inoculum and algal blend inclusion level, and the residual error. Orthogonal polynomial contrasts were used to test the linear and quadratic effects of ruminal inoculum source and the algal blend inclusion level on *in vitro* fermentation parameters. Orthogonal polynomial coefficients for non-equal (ruminal inoculum source) and equal (algal blend level) were computed using the Interactive Matrix Language procedure (SAS OnDemand for Academics). Multiple comparisons of means were carried out using Tukey’s post-hoc test. Significance was set as a *p*-value of <0.05, and a tendency was set at *p* ≥ 0.5 and < 1.0.

## 3 Results

### 3.1 *In vivo* trial

Although dietary treatments were offered in similar amounts to the late-lactation dairy cows (17.5–17.8 kg/day DM; Table 1), the DM intake was greatly affected by the basal forage (*p* < 0.001; Table 3); cows fed corn silage-based diets (100CS and 70CS30HL) had the highest DM intake, followed by those fed 30CS70HL; the lowest DM intake was recorded in cows fed 100% haylage.

**Table 3:** Dry matter intake and ruminal fermentation parameters for cows fed the experimental diets.

	Diets^1^					
	100CS	70CS30HL	30CS70HL	100HL	SEM	*p*
Dry matter intake, kg/day	17.3^c^	17.2^c^	16.3^b^	15.7^a^	0.10	<0.001
Ruminal parameters						
pH	6.54^a^	6.57^a^	6.95^c^	6.78^b^	0.020	<0.001
Ammonia-N, mg/dL	5.81^a^	8.24^b^	10.19^c^	10.96^c^	0.261	<0.001
Total VFA, mmol/L	192	193	181	186	3.3	0.088
Acetate, % mol	62.8^a^	63.3^b^	64.1^c^	64.6^d^	0.09	<0.001
Propionate, % mol	18.4	18.6	17.8	18.4	0.21	0.090
Isobutyrate, % mol	1.13^a^	1.30^b^	1.31^b^	1.32^b^	0.015	<0.001
Butyrate, % mol	14.3^c^	13.3^b^	13.0^b^	11.9^a^	0.29	0.001
Isovalerate, % mol	1.67^a^	1.90^b^	1.95^b^	1.91^b^	0.025	<0.001
Valerate, % mol	1.16^a^	1.15^a^	1.30^b^	1.37^b^	0.018	<0.001
Isocaproate, % mol	0.006	0.017	0.010	0.012	0.0034	0.236
Caproate, % mol	0.491	0.435	0.481	0.481	0.0203	0.281
A: P ratio	3.41^a^	3.40^a^	3.60^b^	3.51^ab^	0.035	0.012
P: B ratio	1.30^a^	1.44^ab^	1.39^ab^	1.54^b^	0.043	0.019
A: (P + B) ratio	1.92^a^	1.99^b^	2.08^c^	2.12^d^	0.009	<0.001

^a,b,c,d^Values in the same row with different superscript letters differ significantly (*p* > 0.05).

^1^Diets are named according to the proportions (dry matter [DM] basis) of corn silage (CS) and haylage (HL) used as the basal forage; 100CS, 100% CS; 70CS30HL, 70% CS and 30% HL; 30CS70HL, 30% CS and 70% HL; 100HL, 100% HL.

VFA, volatile fatty acids; A: P ratio, acetate-to-propionate ratio; P: B ratio, propionate-to-butyrate ratio; A: (P + B) ratio, acetate-to-propionate plus butyrate ratio; SEM, standard error of the mean.

The proportion of corn silage and/or haylage as basal forage also affected most of the ruminal fermentation parameters (Table 3), reflecting the differences in the chemical composition of experimental diets (Table 1). Ruminal pH was lowest in cows fed corn silage-based diets (100CS and 70CS30HL) and was highest in those fed the 30CS70HL diet (*p* < 0.001). The ammonia-N content was highest in cows fed haylage-based diets (30CS70HL and 100HL), followed by those fed 70CS30HL, and was lowest in cows fed 100% corn silage (*p* < 0.001). Dietary treatment tended to affect ruminal total VFA concentrations (*p* = 0.088) and significantly affected the molar proportions and ratios of most of the individual VFA. The molar proportion of acetate gradually increased as the proportion of haylage in the basal forage increased (*p* < 0.001). The proportion of butyrate was highest in cows fed 100CS and lowest in those fed 100HL; cows fed a mixture of corn silage and haylage (70CS30HL and 30CS70HL) had intermediate levels of butyrate (*p* = 0.001). The ruminal molar proportion of valerate was lower in cows fed corn silage-based diets (100CS and 70CS30HL) than in those fed haylage-based diets (30CS70HL and 100HL) (*p* < 0.001). The molar proportions of the branched-chain VFA isobutyrate and isovalerate were lowest when the basal forage contained only corn silage (*p* < 0.001). The experimental diets did not affect propionate (*p* = 0.090), isocaproate (*p* = 0.236), or caproate (*p* = 0.281) proportions. The highest acetate-to-propionate ratio was observed in the ruminal fluid of cows fed the 30CS70HL diet, while the lowest was recorded in cows fed corn silage-based diets (100CS and 70CS30HL) (*p* = 0.012); no difference was observed between 100HL and the other diets. The highest propionate-to-butyrate ratio was recorded with the 100HL diet and the lowest with the 100CS diet (*p* = 0.019); the mixture of basal forages led to intermediate values similar to 100 CS and 100HL. The acetate-to-propionate plus butyrate ratio was lowest with 100% corn silage as the basal forage (100CS) and gradually increased with increasing haylage proportions (*p* < 0.001).

### 3.2 *In vitro* trial

**Figure 1:**
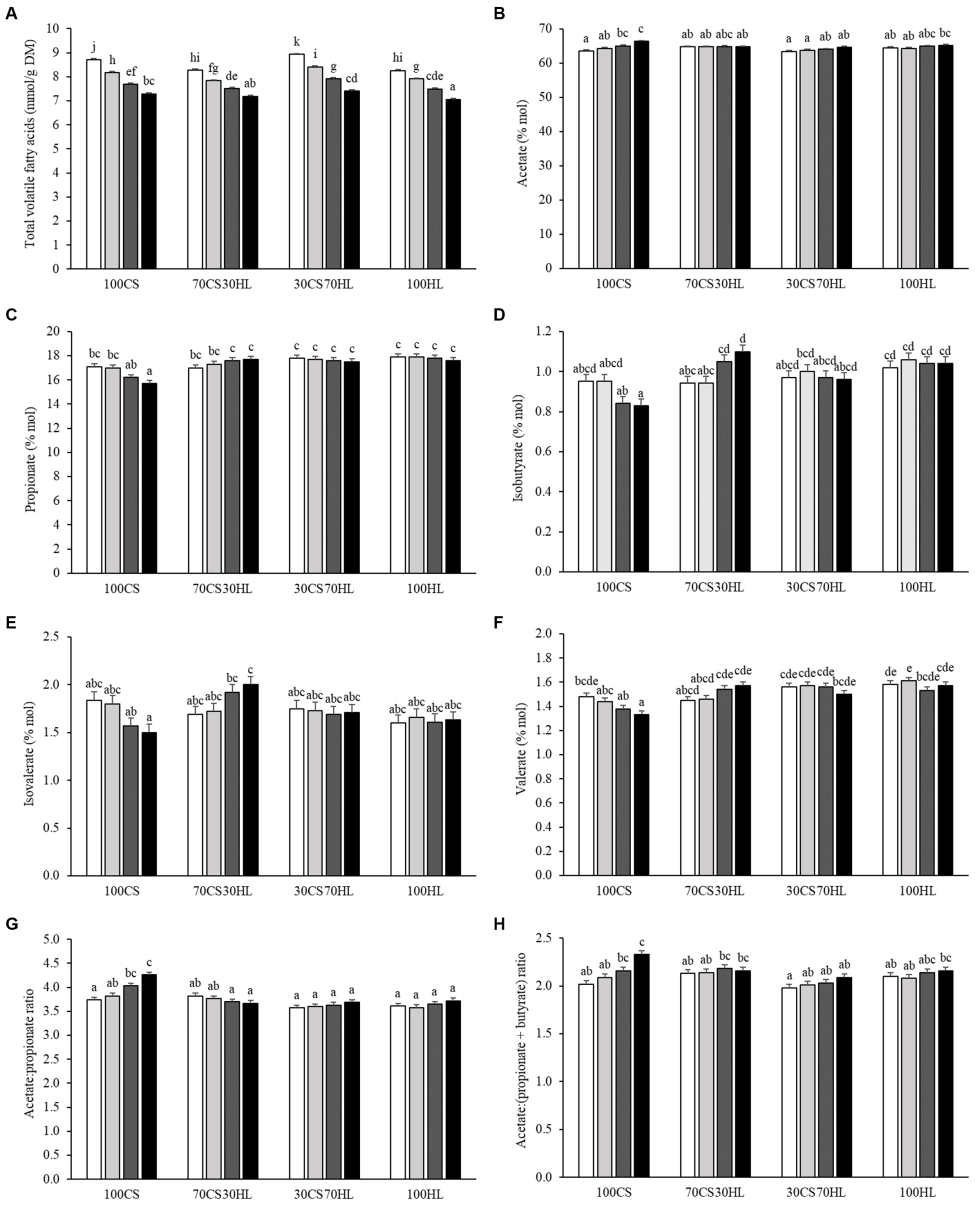
The effect of the interaction between ruminal inoculum derived from forage-based diets and algal blend supplementation levels on **(A)** total volatile fatty acids (mmol/g dry matter [DM]), **(B)** acetate (% mol), **(C)** propionate (% mol), **(D)** isobutyrate (% mol), **(E)** isovalerate (% mol), **(F)** valerate (% mol), **(G)** acetate-to-propionate ratio, and **(H)** acetate-to-propionate plus butyrate ratio after 24 h of *in vitro* incubation. Ruminal inocula are named according to the proportions (DM basis) of corn silage (CS) and haylage (HL) used as basal forage in the experimental diets; 100CS, 100% CS; 70CS30HL, 70% CS and 30% HL; 30CS70HL, 30% CS and 70% HL; 100HL, 100% HL. Algal blend supplementation levels are named according to the proportion (DM basis) of algal blend added to the basal substrate (CS: HL, 1:1); A0, 0% algal blend (control; white bars); A5, 5% algal blend (light grey bars); A10, 10% algal blend (dark grey bars); and A15, 15% algal blend (black bars). ^A–k^ Different superscript letters above the bars denote that the respective mean values differ significantly (*p* < 0.05).

The linear and quadratic effects of the addition of ruminal inocula sourced from the different diets (100CS, 70CS30HL, 30CS70HL, and 100HL) and algal blend supplementation of differing proportions (A0, A5, A10, and A15) to a basal substrate (corn silage and haylage, 1:1 DM basis) on gas and methane production and fermentation parameters after 24 h of *in vitro* incubation are presented in Table 4. The interaction between the ruminal inoculum source and algal blend supplementation level is shown in Supplementary Table S1 and Figure 1. Total gas and methane production followed similar linear (*p* < 0.001) and quadratic (*p* < 0.001) patterns, that is, the highest values were observed with ruminal inocula from cows fed corn silage as the basal forage and the lowest with ruminal inocula from cows fed diets with 70 and 100% haylage (*p* < 0.001; Table 4). In addition, total gas and methane production exhibited a linear decrease (*p* < 0.001) with increasing algal blend supplementation levels. No effect of interaction between the ruminal inoculum source and the algal blend supplementation level was observed for total gas (*p* = 0.332) or methane (*p* = 0.463) production (Supplementary Table S1).

**Table 4:** The effects of ruminal inocula derived from the forage-based diets and algal blend supplementation levels on fermentation parameters after 24 h of *in vitro* incubation.

Parameter	Ruminal inoculum^1^					*p* ^3^		Algal blend^2^					*p* ^3^	
	100CS	70CS30HL	30CS70HL	100HL	SEM	L	Q	A0	A5	A10	A15	SEM	L	Q
Gas, mL/g DM	187	173	168	167	2.0	<0.001	<0.001	183	177	170	166	1.6	<0.001	0.607
Methane, mL/g DM	19.6	17.9	15.8	16.1	0.23	<0.001	<0.001	17.9	17.8	16.9	16.8	0.19	<0.001	0.463
pH	6.08	6.17	6.19	6.20	0.005	<0.001	<0.001	6.16	6.16	6.17	6.16	0.004	0.114	0.366
Ammonia-N, mg/g DM	30.3	26.1	28.4	24.4	0.41	<0.001	0.731	27.7	27.7	27.0	26.8	0.33	0.123	0.660
Total VFA, mmol/g DM	7.97	7.70	8.17	7.68	0.024	0.020	<0.001	8.55	8.08	7.65	7.24	0.020	<0.001	0.237
Acetate, % mol	64.8	64.7	63.9	64.7	0.18	0.185	0.009	64.0	64.2	64.7	65.2	0.21	<0.001	0.388
Propionate, % mol	16.5	17.4	17.7	17.8	0.14	<0.001	0.002	17.4	17.5	17.3	17.1	0.12	0.033	0.284
Isobutyrate, % mol	0.893	1.01	0.976	1.04	0.0193	<0.001	0.140	0.969	0.988	0.976	0.983	0.0158	0.691	0.701
Butyrate, % mol	14.2	13.0	13.7	12.8	0.28	0.016	0.561	13.8	13.5	13.3	13.1	0.22	0.016	0.977
Isovalerate, % mol	1.68	1.83	1.72	1.63	0.049	0.283	0.003	1.72	1.73	1.70	1.71	0.040	0.777	0.950
Valerate, % mol	1.41	1.50	1.55	1.57	0.017	<0.001	0.012	1.52	1.52	1.50	1.49	0.014	0.122	0.753
Isocaproate, % mol	0.0183	0.0207	0.0228	0.0230	0.00207	0.129	0.505	0.0187	0.0229	0.0227	0.0206	0.00169	0.471	0.067
Caproate, % mol	0.494	0.541	0.416	0.503	0.0101	0.230	0.019	0.520	0.505	0.474	0.455	0.0082	<0.001	0.782
A: P ratio	3.96	3.74	3.62	3.64	0.033	<0.001	<0.001	3.68	3.69	3.75	3.83	0.027	<0.001	0.153
P: B ratio	1.17	1.40	1.29	1.40	0.031	0.210	0.024	1.28	1.31	1.33	1.34	0.026	0.110	0.804
A: (P + B) ratio	2.15	2.15	2.02	2.12	0.022	0.042	0.013	2.06	2.08	2.13	2.18	0.018	<0.001	0.348

^1^Ruminal inocula are named after experimental diets according to the proportions (dry matter [DM] basis) of corn silage (CS) and haylage (HL) used as basal forage; 100CS, 100% CS; 70CS30HL, 70% CS and 30% HL; 30CS70HL, 30% CS and 70% HL; 100HL, 100% HL.

^2^Supplementation levels are named according to the proportion (DM basis) of algal blend added to the basal substrate (CS: HL, 1:1): A0, 0% algal blend (control); A5, 5% algal blend; A10, 10% algal blend; A15, 15% algal blend.

L, linear orthogonal polynomial contrast; Q, quadratic orthogonal polynomial contrast; VFA, volatile fatty acids; A: P ratio, acetate-to-propionate ratio; P: B ratio, propionate-to-butyrate ratio; A: (P + B) ratio, acetate-to-propionate plus butyrate ratio; SEM, standard error of the mean.

Fermentation pH was only affected by ruminal inoculum source (*p* < 0.001; Table 4), increasing linearly and quadratically (*p* < 0.001) when corn silage was replaced by haylage as the basal forage in the diets of ruminal inocula donors. Ammonia-N contents displayed a linear decrease (*p* < 0.001) in fermentation media following incubation with inocula from cows fed diets containing increasing haylage replacement levels; no linear (*p* = 0.123) or quadratic (*p* = 0.660) effects on ammonia-N contents were observed for the algal blend inclusion level (Table 4) or ruminal inoculum source and algal blend supplementation level interaction (*p* = 0.660; Supplementary Table S1).

Total VFA production and the molar proportions and ratios of most individual VFA were affected (*p* < 0.05) by the interaction between the ruminal inoculum source and the algal blend supplementation level (Supplementary Table S1 and Figure 1). After 24 h of *in vitro* incubation, total VFA production decreased with increasing algal blend supplementation levels with all inoculum sources (*p* < 0.001); the lowest production was found when A15 was incubated with the 100HL inoculum, while the highest VFA production was observed with incubation of A0 with the 30CS70HL inoculum. Compared with the control (A0), algal blend supplementation at the 10 and 15% levels increased the molar proportion of acetate under incubation with 100CS (*p* = 0.009); no differences were observed among algal blend supplementation levels when incubated with inocula from other sources. The molar proportion of propionate was lower with A15 than with A0 and A10 supplementation when the 100CS inoculum was used (*p* = 0.006), but not under incubation with inocula derived from cows fed haylage-containing diets. Isobutyrate (*p* = 0.001) and isovalerate (*p* = 0.018) molar proportions followed similar patterns, with the lowest and highest contents being recorded with the 15% algal blend supplementation level under incubation with the 100CS and 70CS30HL inocula, respectively. The molar proportion of valerate was lower with the A15 than the A0 supplementation level with the 100CS inoculum (*p* = 0.001); no differences were observed among algal supplementation levels with other inoculum sources. The acetate-to-propionate ratio was higher with the A10 and A15 supplementation levels than when no algal blend was added (A0) under incubation with the 100CS inocula (*p* < 0.001); no differences were detected among algal blend supplementation levels with other inoculum sources. The lowest acetate-to-propionate plus butyrate ratio was recorded with the A0 inclusion level under incubation with the 30CS70HL inoculum and the highest was observed with the A15 inclusion level under incubation with the 100CS inoculum (*p* = 0.041).

Conversely, the interaction between the ruminal inoculum source and the algal blend supplementation level did not affect butyrate (*p* = 0.922) and caproate (*p* = 0.249) proportions or the propionate-to-butyrate ratio (*p* = 0.809) but tended to affect the isocaproate proportion (*p* = 0.050; Supplementary Table S1). The molar proportion of butyrate exhibited a linear decrease with increasing haylage proportions in the diets of ruminal inoculum donors (*p* = 0.016) and algal blend supplementation levels (*p* = 0.016; Table 4). No linear or quadratic effect of inoculum source (*p* = 0.129 and 0.505) or algal blend supplementation level (*p* = 0.471 and 0.067) was observed on the isocaproate proportion (Table 4). Ruminal inoculum source had a quadratic effect on the caproate molar proportion (*p* = 0.019), that is, caproate proportions were highest under incubation with the 70CS30HL inoculum and lowest under incubation with the 30CS70HL inoculum; meanwhile, increasing algal blend supplementation levels led to a linear decrease in caproate proportion (*p* < 0.001; Table 4). The ruminal inoculum source exerted a quadratic effect on the propionate-to-butyrate ratio (*p* = 0.024), with the lowest value found in fermentation medium incubated with the 100CS inoculum and the highest values observed in fermentation medium containing the 70CS30HL or the 100HL inoculum (Table 4).

## 4 Discussion

*In vitro* rumen batch culture is a technique widely used for predicting the degradability and nutritive value of feed sources (23, 24). It is also used to screen the potential of non-conventional additives and feeds, such as biochars, microalgae, and seaweeds (6, 25, 26), to modulate ruminal fermentation and, in particular, to reduce methane production. Despite its simplicity and the possibility of testing multiple samples at a single batch under similar conditions and without interference from the metabolic process of the host, several factors may influence the consistency of results (11, 23). Among these factors, the ruminal inoculum plays a major role.

Variations in *in vitro* gas production and fermentability due to the ruminal inoculum have been attributed to differences in the microbiota population profile and activity, among and within donor animals, between days, in sampling times (before or after feeding), and in the dietary composition and nutrient and energy intake of the host animals (7, 8, 27). In our study, we focused on the impact of the composition of the basal forage of diets on the ruminal inoculum of late-lactation cannulated cows. Four diets were formulated with similar ingredients, varying only in the proportions of two types of basal forage of differing chemical composition and particle size. Proper forage particle size is crucial for normal ruminal function and host health as it promotes a stable digesta mat, which increases the retention time of fiber particles, thereby promoting their fermentation by the rumen microbial community and stimulating reticulorumen motility, rumination, and salivation, neutralizing excessive acid production, and preventing a pH drop below levels required for optimal fermentation (28, 29). Along with other factors, particle size also plays an important role in feed intake and, thus, in dietary nutrient and energy digestibility. In the current study, a decrease in DM intake was observed when cows were fed diets with increasing proportions of haylage in substitution of corn silage, with cows fed the 100HL diet consuming 1.6 kg/day less than animals fed the 100CS diet. These differences may be due to the longer time required for eating and chewing haylage-containing diets, greater rumen fill effects, and longer retention times (30). Indeed, one study noted that, in early-lactating cows, the DM intake was reduced by 2.8 and 3.5 kg/day with 50% replacement of corn silage by long chop and short chop ryegrass silage, respectively (31). In contrast, a different study reported that the replacement of 0, 35, 65, and 100% ryegrass silage with corn silage had no impact on DM intake in cows during late lactation (32). These contrasting results highlight the importance of silage chop length on feed intake and performance (31).

Changing the acidogenicity value and the physically effective neutral detergent fiber contents of diets are complementary dietary formulation strategies for effectively manipulating the ruminal acid load (14, 33), with the carbohydrate type (starch vs. fiber) greatly influencing the ruminal microbial population and the fermentation profile (29, 34, 35). In the present study, these changes were reflected in the alterations in the ruminal fluid fermentation profiles observed when corn silage was replaced with haylage and in the concomitant increase in the neutral detergent fiber-to-starch ratio (1.8 to 10.5). These changes resulted in increases in pH values, the acetate molar proportion, and the acetate-to-propionate plus butyrate ratio, consistent with a higher fiber content (36). The ammonia-N content was 1.9 times higher in the ruminal fluid of cows fed 100HL than in that of cows fed 100CS, reflecting the higher crude protein content of the 100HL diet, further suggesting that corn silage-based diets improve the synchronization of energy and N release [synchronism concept, (37)]. Indeed, fermentable carbohydrates have been shown to reduce ammonia production by enhancing the capture of released ammonia-N or amino acids by ruminal microbiota or by reducing amino acid deamination (38, 39). While the total VFA content reflects the ruminal fermentation efficiency, the dietary treatment showed only a tendency (*p* = 0.088) to affect ruminal total VFA concentrations in the current study. The similar total VFA concentrations found may have been due to the ruminal fluid being collected before the morning meal when VFA production is lower (40) and the microbiota population is more stable (34).

High biomass productivity, a low carbon and water footprint, and significant nutritive and functional values have influenced the interest in the use of algae as a non-conventional and sustainable animal feed. Several studies have assessed the effects of microalgal and seaweed species as supplements and/or ingredients for ruminant feeding [see reviews (41, 42)]. Recently, the combination of microalgae (43, 44) and of seaweeds (45, 46) was reported to improve ruminant nutrition and milk quality. However, to the best of our knowledge, only one recent study evaluated the effects of a mixture of one microalgal (*Euglena gracilis*) and one seaweed (*Asparagopsis taxiformis*) species on *in vitro* ruminal fermentation parameters (47), and none of the studies have investigated the potential effects of combining two microalgal and two seaweed species. Thus, in this study, we assessed, for the first time, the effects of increasing supplementation levels (0, 5, 10, and 15%, DM basis) of a commercially available algal blend composed of two microalgal and two seaweed species on gas and methane production, pH, ammonia-N contents, and total VFA production and the proportion of individual VFA after 24 h of incubation with ruminal inocula obtained from cows fed forage-based diets with different proportions of two basal forages. A wide range of algal supplementation levels are reported in the literature, with microalgae being included at lower levels (up to 10%, 26) and seaweeds at higher levels (up to 25%, 6, 15, 47). As the commercially available algal blend used in the present study was composed of both microalgae and seaweeds, low (5%), average (10%), and high (15%) supplementation levels were assessed to gain further insights into the potential of this non-conventional and sustainable feed in ruminant nutrition.

In short-term *in vitro* systems such as batch culture technique, the microbiota of the ruminal inoculum cannot adapt to the substrate provided; thus, the rate, extent, and parameter profile of fermentation greatly depend on the composition and activity of the microbial population in the ruminal inoculum. Consequently, although not always feasible, the current recommendations are that the basal substrate should be similar to that in the diets of the donor animals (8, 11). Thus, in the present study, the basal substrate used was a combination (1:1) of the two base forages (corn silage and haylage) of the diets of the ruminal content donors supplemented with increasing algal blend levels; following incubation, *in vitro* fermentability was evaluated. The ruminal inoculum source was found to have a stronger impact than algal blend inclusion levels of up to 15% (DM basis) on short-term *in vitro* fermentation parameters. Inocula from cows fed diets in which corn silage was replaced with haylage effectively reduced total gas and methane production by 10.7 and 17.9%, respectively, while increasing the algal blend supplementation level contributed to reductions of 9.3 and 6.2%, respectively. Methane is the predominant greenhouse gas produced by ruminants. In addition to a high energetic cost for the animal, methane production is associated with a high environmental burden (48); indeed, it has been estimated that to limit the global temperature increase to 1.5°C, it is necessary to decrease enteric methane emissions by 20% by 2030, using 2020 as the baseline (49). In addition to reducing methane production, the algal blend used in this study is a sustainable feed as microalgae and seaweeds are produced autotrophically in local photobioreactors and IMTA systems, respectively, in which energy is derived from solar power, and nutrients are obtained from fish aquaculture wastewaters (50, 51). However, owing to the recalcitrant nature of the cell walls of microalgae and seaweeds, the fermentability of the algal blend may have been impaired, as complex algal cell wall polysaccharides are absent in conventional feeds and the microbial populations of the ruminal inocula were not adapted to this unconventional feed source (6, 26). Meanwhile, the effect of the ruminal inoculum source was greater than expected. Although studies have reported great effects of ruminal inocula source on fermentation rates, the extent of fermentation, and, thus, gas production, is often not affected, or only mildly so (52). In addition, in a review of the factors that affect *in vitro* gas production, Rymer et al. (27) concluded that above a minimum activity level of the microbiota, the ruminal inoculum source did not affect gas production, as long as a diet containing forage and concentrate, ideally in a 60:40 ratio, was fed to the ruminal fluid donors. Even though such a forage-based diet was provided to the ruminal fluid donor animals in the current study, microbial population activity may have been affected as the ammonia-N concentrations after 24 h of incubation was below the minimum level required for efficient microbial production and cellulolytic activity [50 mg/L; (53)].

In this study, the interaction between ruminal inoculum source and algal blend supplementation level affected total VFA production and the proportions of most individual VFA. Total VFA production was found to decrease with increasing algal supplementation levels with all inocula, with the strongest reduction being observed with inocula from cows fed the 30CS70HL diet. Given that VFA are the primary energy source of the ruminant host (54), feed sources with methane mitigation potential that do not reduce ruminal fermentation and total VFA production are of particular interest (49, 55). Acetate, propionate, and butyrate are the main VFA produced in the rumen, and their ratios mainly reflect the fermentation of non-structural and structural polysaccharides. In the current study, the molar proportions of acetate and propionate were, respectively, higher and lower after 24 h of incubation with the algal blend at the 15% supplementation level compared with those observed with the 0 and 5% inclusion levels, but only when the inoculum was derived from cows fed 100% corn silage as the base forage; no interaction effect was observed for butyrate. Higher acetate and butyrate and lower propionate production have been associated with increased gas production (56) and thus also with enhanced fermentability and microbiota production and activity; however, such an association was not observed in our study. The supplementation of corn silage or haylage substrate with microalgal (26) or macroalgal (6) species resulted in different fermentation profiles, with haylage showing lower fermentability. As previously mentioned, diet is known to greatly affect the composition and activity of the ruminal microbiota. Although changes in the ruminal microbiota can partly explain the results obtained in our study, Jami and Mizrahi (57) reported that differences in ruminal bacterial populations among animals fed the same diet were substantially higher than those promoted by different diets. Moreover, the authors concluded that despite the variation in bacterial taxa among animals, the microbiota was phylogenetically highly similar (82%) and thus did not affect fermentation ability. Hua et al. (58) compared the effects of glucogenic and lipogenic diets on ruminal bacterial community structure, fermentation intermediary metabolites, and methane production following 48 h of incubation with a ruminal inoculum. Although several amylolytic and cellulolytic bacteria were found to be sensitive to dietary differences, most highly abundant bacteria were stable or were only marginally affected, thus highlighting the relevance of the microbiota profile of ruminal inocula in short-term *in vitro* fermentation studies.

## 5 Conclusion

Ruminal inoculum source markedly affected the *in vitro* fermentability of the algal blend, supporting the importance of the diet of rumen donors in the modulation of the microbial ecosystem and its impact on the *in vitro* evaluation of non-conventional feeds for ruminants. Indeed, the replacement of corn silage with haylage as the basal forage in the diet of late-lactation dairy cows decreased gas and methane production and modified the ruminal fermentation profile after 24 h of *in vitro* batch incubation. Gas and methane production was also reduced with algal blend supplementation levels of up to 15% (DM basis). The interaction between the ruminal inoculum source and the algal blend supplementation level exerted an effect only on total VFA production and the profiles of most individual VFA; this observation highlights the need to consider the diet of ruminal inoculum donors and the algal blend inclusion level when evaluating the optimal supplementation strategy for promoting ruminant nutrition and production sustainability without impairing ruminal function. Overall, our results highlight the importance of ruminal inocula when assessing the fermentability of a non-conventional feed. Our findings further highlight the potential of the algal blend as a natural modulator of ruminal fermentation *in vitro*; however, further *in vivo* studies are needed to reveal its full potential.

## Data availability statement

The original contributions presented in the study are included in the article/Supplementary material, further inquiries can be directed to the corresponding author.

## Ethics statement

All procedures involving animals were approved by the Animal Ethics Committee of the School of Medicine and Biomedical Sciences, University of Porto (ICBAS-UP) and licenced by the Portuguese General Directorate of Food and Veterinary Affairs (permit #0421/000/000/2021). All the procedures were performed by trained scientists (FELASA category C) in accordance with the European Union Directive 2010/63/EU relating to the protection of animals used for scientific purposes.

## Author contributions

CM: Formal analysis, Investigation, Writing – original draft, Writing – review & editing. MM: Formal analysis, Investigation, Writing – original draft, Writing – review & editing, Conceptualization, Validation. IV: Investigation, Writing – review & editing, Conceptualization. AC: Formal analysis, Writing – review & editing, Conceptualization. AF: Funding acquisition, Writing – original draft, Writing – review & editing, Conceptualization, Resources, Supervision, Validation.
