# Supplementary material for: Ruminal inocula with distinct fermentation profiles differentially affect the in vitro fermentation pattern of a commercial algal blend
Source: Front Vet Sci. 2024 Mar 11;11:1346683. doi: 10.3389/fvets.2024.1346683 (PMC10962210; doi:10.3389/fvets.2024.1346683)
Supplement: Supplementary file 1 [file Table_1.DOCX]

Supplementary Material

Ruminal inocula with distinct fermentation profiles differentially affect the *in vitro* fermentation pattern of a commercial algal blend

Cátia S. C. Mota^1^, Margarida R. G. Maia^1*^_,_ Inês M. Valente^1,2^, Ana R. J. Cabrita^1^, António J. M. Fonseca^1^

^1^REQUIMTE, LAQV, ICBAS, School of Medicine and Biomedical Sciences, University of Porto, Rua Jorge Viterbo Ferreira 228, 4050-313 Porto, Portugal

^2^REQUIMTE, LAQV, Department of Chemistry and Biochemistry, Faculty of Sciences, University of Porto, Rua do Campo Alegre 687, 4169-007 Porto, Portugal

*** Correspondence:**Margarida R. G. Maia
[mrmaia@icbas.up.pt](mailto:mrmaia@icbas.up.pt)

**Table S1.** Effect of the interaction between rumen inocula derived from the forage-based diets^1^ and algae blend supplementation levels^2^ on fermentation parameters after 24-h *in vitro* incubation.

|  | **100CS** | | | | **70CS30HL** | | | | **30CS70HL** | | | | **100HL** | | | |  |  |
| --- | --- | --- | --- | --- | --- | --- | --- | --- | --- | --- | --- | --- | --- | --- | --- | --- | --- | --- |
|  | **A0** | **A5** | **A10** | **A15** | **A0** | **A5** | **A10** | **A15** | **A0** | **A5** | **A10** | **A15** | **A0** | **A5** | **A10** | **A15** | **SEM** | ***p*** |
| Gas, mL/g DM | 196 | 193 | 180 | 179 | 178 | 177 | 173 | 165 | 176 | 170 | 166 | 162 | 182 | 168 | 161 | 158 | 3.4 | 0.332 |
| Methane, mL/g DM | 19.7 | 20.8 | 19.0 | 19.0 | 18.4 | 18.0 | 17.8 | 17.3 | 16.3 | 15.8 | 15.5 | 15.4 | 17.0 | 16.6 | 15.4 | 15.4 | 0.39 | 0.463 |
| pH | 6.09 | 6.08 | 6.09 | 6.08 | 6.17 | 6.16 | 6.18 | 6.17 | 6.18 | 6.18 | 6.21 | 6.19 | 6.20 | 6.20 | 6.20 | 6.20 | 0.008 | 0.366 |
| Ammonia-N, mg/g DM | 31.8 | 30.4 | 29.7 | 29.3 | 26.1 | 26.0 | 26.3 | 26.0 | 28.7 | 29.1 | 28.0 | 27.8 | 24.3 | 25.3 | 24.0 | 24.2 | 0.71 | 0.660 |
| Total VFA, mmol/g DM | 8.72^j^ | 8.17^h^ | 7.69^ef^ | 7.29^bc^ | 8.28^hi^ | 7.83^fg^ | 7.51^de^ | 7.19^ab^ | 8.93^k^ | 8.41^i^ | 7.92^g^ | 7.41^cd^ | 8.26^hi^ | 7.91^g^ | 7.49^cde^ | 7.06^a^ | 0.042 | <0.001 |
| Acetate, % mol | 63.5^a^ | 64.3^ab^ | 65.0^bc^ | 66.3^c^ | 64.7^ab^ | 64.7^ab^ | 64.8^abc^ | 64.7^ab^ | 63.4^a^ | 63.7^a^ | 64.0^ab^ | 64.6^ab^ | 64.5^ab^ | 64.2^ab^ | 64.9^abc^ | 65.1^bc^ | 0.32 | 0.009 |
| Propionate, % mol | 17.1^bc^ | 17.0^bc^ | 16.2^ab^ | 15.7^a^ | 17.0^bc^ | 17.3^bc^ | 17.6^c^ | 17.7^c^ | 17.8^c^ | 17.7^c^ | 17.6^c^ | 17.5^c^ | 17.9^c^ | 17.9^c^ | 17.8^c^ | 17.6^c^ | 0.24 | 0.006 |
| Isobutyrate, % mol | 0.952^abcd^ | 0.952^abcd^ | 0.841^ab^ | 0.829^a^ | 0.934^abc^ | 0.943^abc^ | 1.05^cd^ | 1.10^d^ | 0.971^abcd^ | 1.00^bcd^ | 0.971^abcd^ | 0.961^abcd^ | 1.02^cd^ | 1.06^cd^ | 1.04^cd^ | 1.04^cd^ | 0.0334 | 0.001 |
| Butyrate, % mol | 14.5 | 14.0 | 14.5 | 13.8 | 13.7 | 13.4 | 12.5 | 12.5 | 14.1 | 13.8 | 13.7 | 13.3 | 12.9 | 13.0 | 12.6 | 12.6 | 0.48 | 0.922 |
| Isovalerate, % mol | 1.84^abc^ | 1.80^abc^ | 1.57^ab^ | 1.50^a^ | 1.69^abc^ | 1.72^abc^ | 1.92^bc^ | 2.00^c^ | 1.75^abc^ | 1.73^abc^ | 1.69^abc^ | 1.71^abc^ | 1.60^abc^ | 1.66^abc^ | 1.61^abc^ | 1.63^abc^ | 0.085 | 0.018 |
| Valerate, % mol | 1.48^bcde^ | 1.44^abc^ | 1.38^ab^ | 1.33^a^ | 1.45^abcd^ | 1.46^abcd^ | 1.54^cde^ | 1.57^cde^ | 1.56^cde^ | 1.57^cde^ | 1.56^cde^ | 1.50^bcde^ | 1.58^de^ | 1.61^e^ | 1.53^bcde^ | 1.57^cde^ | 0.030 | 0.001 |
| Isocaproate, % mol | 0.014 | 0.016 | 0.023 | 0.021 | 0.022 | 0.030 | 0.017 | 0.015 | 0.014 | 0.026 | 0.026 | 0.025 | 0.026 | 0.020 | 0.024 | 0.022 | 0.0036 | 0.050 |
| Caproate, % mol | 0.535 | 0.521 | 0.467 | 0.455 | 0.542 | 0.539 | 0.553 | 0.531 | 0.453 | 0.436 | 0.400 | 0.376 | 0.548 | 0.526 | 0.478 | 0.460 | 0.0175 | 0.249 |
| A:P ratio | 3.74^a^ | 3.82^ab^ | 4.03^bc^ | 4.26^c^ | 3.82^ab^ | 3.76^ab^ | 3.70^a^ | 3.67^a^ | 3.57^a^ | 3.60^a^ | 3.63^a^ | 3.69^a^ | 3.61^a^ | 3.58^a^ | 3.65^a^ | 3.72^a^ | 0.057 | <0.001 |
| P:B ratio | 1.17 | 1.21 | 1.14 | 1.18 | 1.31 | 1.37 | 1.46 | 1.47 | 1.26 | 1.28 | 1.29 | 1.32 | 1.39 | 1.39 | 1.41 | 1.39 | 0.054 | 0.809 |
| A:(P+B) ratio | 2.02^ab^ | 2.09^ab^ | 2.16^bc^ | 2.33^c^ | 2.13^ab^ | 2.14^ab^ | 2.18^bc^ | 2.16^bc^ | 1.98^a^ | 2.01^ab^ | 2.03^ab^ | 2.09^ab^ | 2.10^ab^ | 2.08^ab^ | 2.14^abc^ | 2.16^abc^ | 0.038 | 0.041 |

^a,b,c,d^Values in the same row that share a common superscript are not statistically different (*p* > 0.05).

^1^Rumen inocula are named after experimental diets according to proportions (dry matter [DM] basis) of corn silage (CS) and haylage (HL) as basal forage: 100CS, 100% CS; 70CS30HL, 70% CS and 30% HL; 30CS70HL, 30% CS and 70% HL; 100HL, 100% HL.

^2^Supplementation levels are named according to proportions (DM basis) of algae blend added to basal substrate (CS:HL, 1:1): A0, 0% algae blend (control); A5, 5% algae blend; A10, 10% algae blend; A15, 15% algae blend.

VFA, volatile fatty acids; A: P ratio, acetate-to-propionate ratio; P: B ratio, propionate-to-butyrate ratio; A: (P+B) ratio, acetate-to-propionate plus butyrate ratio; SEM, standard error of the mean.
